# Hollow TiO_2_/Poly (Vinyl Pyrrolidone) Fibers Obtained via Coaxial Electrospinning as Easy-to-Handle Photocatalysts for Effective Nitrogen Oxide Removal

**DOI:** 10.3390/polym14224942

**Published:** 2022-11-15

**Authors:** Juran Kim

**Affiliations:** Advanced Textile R&D Department, Republic of Korea Institute of Industrial Technology (KITECH), Ansan 15588, Republic of Korea; jkim0106@kitech.re.kr

**Keywords:** photocatalyst, titanium dioxide, core-sheath hollow fibers, nitrogen oxide removal

## Abstract

Herein, we present a method for fabricating hollow TiO_2_ microfibers from Ti (OBu)_4_/poly (vinyl pyrrolidone) sol-gel precursors and their effects on denitrification as a photocatalyst for air purification. Various sizes of hollow TiO_2_ fibers were developed using coaxial electrospinning by controlling the core flow rate from 0 to 3 mL h^−1^. At higher flow rates, the wall layer was thinner, and outer and core diameters were larger. These features are correlated with physical properties, including specific surface area, average pore diameter, and crystalline structure. The increase in the core flow rate from 0 to 3 mL h^−1^ leads to a corresponding increase in the specific surface area from 1.81 to 3.95 µm and a decrease in the average pore diameter from 28.9 to 11.1 nm. Furthermore, the increased core flow rate results in a high anatase and rutile phase content in the structure. Herein, hollow TiO_2_ was produced with an approximately equal content of anatase/rutile phases with few impurities. A flow rate of 3 mL h^−1^ resulted in the highest specific surface area of 51.28 m^2^ g^−1^ and smallest pore diameter size of ~11 nm, offering more active sites at the fiber surface for nitrogen oxide removal of up to 66.2% from the atmosphere.

## 1. Introduction

Nitrogen oxides such as NO and NO_2_ (which are commonly known as NO_x_), are major air pollutants primarily emitted during low-grade fossil fuel combustion [1]. NO_x_ causes severe environmental and human health issues, such as respiratory diseases, lung cancer, global warming, ozone production, acid rain, and smog [2]. According to the World Health Organization guidelines, the upper limit of the allowed NO concentration is 40 μg m^−3^ [3].

Recently, many researchers have reported the need for highly effective photocatalysts targeting NO_x_. Owing to the increased attention toward denitrification, titanium dioxide has been extensively used as a photocatalyst for air purification because of its relatively low cost, nontoxicity, easy handling, and long-term stability [3]. TiO_2_ can generate electron–hole (*e^−^*/*h^+^*) pairs at its surface in the presence of ultraviolet (UV) light energy higher than its band gap energy of ~3.2 eV. The exited *e^−^*/*h^+^* pairs then react with H_2_O and O_2_ molecules in the atmosphere, leading to the formation of hydroxy radicals and superoxide oxygen via reactive oxygen species (ROS) [3,4]. Subsequently, these molecules can react with nitrogen oxides to form nitrates via photocatalytic oxidation.

However, brittleness and handling issues of TiO_2_ nanoparticles are the major factors that restrict their applications as photocatalysts for air purification [5]. Therefore, it remains a challenge to develop a facile fabrication method to generate mesoporous TiO_2_ as an effective air pollutant photocatalyst.

Electrospinning is an effective fabrication method to address the aforementioned limitations of TiO_2_ nanoparticles because it provides a simple method to prepare ultrathin TiO_2_ fibers with diameters ranging from several nano- to micrometers. Polymer, organic, inorganic, and hybrid core/shell or hollow materials have been fabricated via coaxial electrospinning, in which a spinneret consisting of two coaxial capillaries form a core-sheath structure [6]. Controlling the variables using coaxial electrospinning can modify the architecture and multifunction of fibers.

Core-sheath or hollow materials have attracted extensive attention in recent years because of their unique heterogeneous or hollow structures that offer high surface-to-volume ratios and core loading spaces, rendering them useful for air purification [6]. For example, Chen et al. reported a multifluidic coaxial electrospinning method to fabricate photocatalytic TiO_2_ nanowire-in-microtube structured nanofibers [7]. The mesoporous TiO_2_ hollow nanofibers, which were fabricated via a facile single-capillary electrospinning technique, showed 99.5% degradation of Rhodamine B within 60 min, suggesting their promising application as efficient photocatalysts [8]. Additionally, polyaniline/TiO_2_ bilayer microtubes with an average diameter of 200 nm were fabricated for photocatalysis [9]. Polyvinylidene fluoride (PVDF)–TiO_2_ composite hollow fibers were prepared using the sol-gel method [10]. Furthermore, fluorinated titania-silica/PVDF hollow fibers were developed for CO_2_ removal [11]. Electrospinning was used to prepare PVDF/poly dimethylsiloxane composites with TiO_2_ nanoparticles (0.5 and 1 wt.%) for NOx adsorption [12].

Herein, coaxial electrospinning was used under four types of processing conditions to develop hollow TiO_2_ fibers as photocatalysts for air purification. An ideal hollow architecture with a micro-nanoporous structure was achieved for excellent photocatalytic performance. Subsequently, physical properties, including morphology, specific surface area, pore diameter, and crystalline phase, of four types of TiO_2_ fibers were evaluated. Moreover, their photocatalytic performance was evaluated for denitrification under UV irradiation. Additionally, the physical properties of hollow TiO_2_ fibers were correlated to the photocatalytic performance. This study provides a comprehensive understanding and efficient design approach toward hollow TiO_2_ fibers for various applications, such as surface coating, electronics, biomedicine, sensing, and water and air purification.

## 2. Materials and Methods

### 2.1. Materials

Poly(vinyl pyrrolidone) (PVP) (Mw ≈ 130,000), tetrabutyl titanate [Ti(OBu)_4_], Tween 80, paraffin oil, ethanol, acetic acid, were purchased from Sigma Aldrich Co., LLC, (Seoul, Republic of Korea).

### 2.2. Preparation of Core-Sheath Sol-Gel

PVP was dissolved in a mixture of ethanol solution and acetic acid (1:8:1 *w*/*w*/*w*) in a flask, followed by the gradual addition of 40 wt.% Ti (OBu)_4_ solution under stirring at room temperature for 4 h to form a homogeneous sol for outer sheath material, such as the Ti (OBu)_4_ sol. For the core material, paraffin oil solution was prepared by dissolving 5 wt.% Tween 80 in 2 wt.% deionized water. Subsequently, the emulsion was stirred for 3 h at room temperature.

### 2.3. Fabrication of Hollow TiO_2_ Fibers

**Figure 1:**
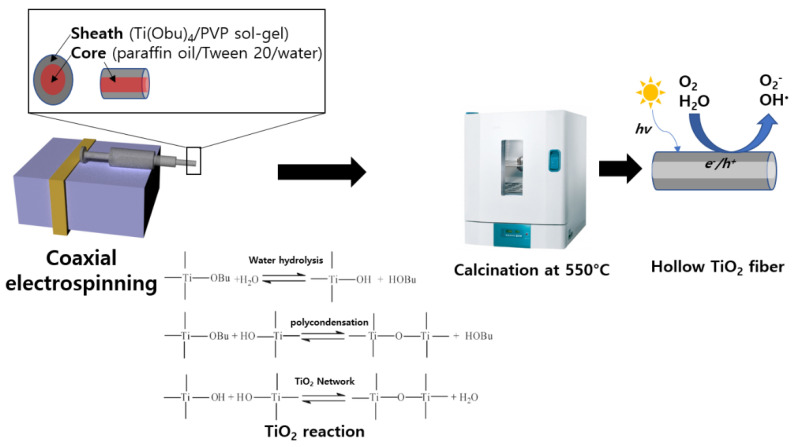
Fabrication method of hollow TiO_2_ fibers via coaxial electrospinning and their reactions for the hydrolysis process with titanium butoxide as a precursor.

The coaxial electrospinning experimental setup is illustrated in Figure 1. The spinneret was assembled using coaxial stainless steel with two capillaries. Typically, the outer and inner diameters were 3.0 and 2.5 mm and 0.6 and 0.37 mm, respectively. The Ti (OBu)_4_ sol synthesized previously was injected into the outer capillaries at a flow rate of 8 mL h^−1^, and the paraffin oil emulsion was simultaneously fed into the inner capillary at 0, 1, 2, or 3 mL h^−1^. The work distance between the spinneret and collector was 15–20 cm, and the work voltage was 25–30 kV. After coaxial electrospinning, the collected fibers were calcinated at 550 °C for 1 h at a heating rate of 1 °C min^−1^. The core was removed after calcination treatment (MF2-12GF, JeioTech, Daegeon, Republic of Korea) to obtain hollow TiO_2_ fibers [13].

On applying a suitable high voltage to the coaxial spinneret, the conductive outer fluids (Ti (OBu)_4_/PVP sol-gel) were elongated owing to an electrostatic force, while the inner fluids (paraffin oil/Tween 20/water) were subsequently stretched because of shear forces. The outer Ti (OBu)_4_ sol-gel solidified easily during the coaxial electrospinning process because of the rapid evaporation of the ethanol solvent and the hydrolysis of metal alkoxide at appropriate humidity [7]. As shown in Figure 1, water is emulsified in paraffin oil to accelerate the hydrolysis and solidification of the core material during reactions [7]. If the core was selectively removed by calcination, then it would leave a vacant space to develop hollow TiO_2_ fibers. Electrospinning was used to fabricate hollow and solid (non-hollow) TiO_2_ fibers under different conditions and they were labelled with relevant codes, as listed in Table 1.

### 2.4. Chemical Structures and Physical Properties of TiO_2_ Fibers

The morphologies and average outer or core diameters of TiO_2_@C1, TiO_2_@C2, TiO_2_@C3, and TiO_2_@C0 were analyzed via scanning electron microscopy (SEM, Hitachi, Tokyo, Japan). For cross sections, samples were immersed in liquid nitrogen and then cut with a razor blade. The N_2_ adsorption and desorption analyses were performed (BELSORP-Mini II, Osaka, Japan) at 77 K. The specific surface areas were calculated from the obtained data using the Brunauer–Emmett–Teller (BET) method. The total pore volume was estimated from the amount adsorbed at a *P*/*P*_0_ of 0–0.99. The average pore diameter was derived using the Barret–Joyner–Halenda (BJH) model. The specific surface areas and pore diameters of TiO_2_@C1, TiO_2_@C2, TiO_2_@C3, and TiO_2_@C0 were evaluated using the BELSORP analysis software.

### 2.5. Characterization of Synthesized TiO_2_ Fibers

X-ray diffraction (XRD, D8 Advance, Bruker, Billerica, MA, USA) was used to analyze the crystalline phases of synthesized TiO_2_ fibers. The diffractometer was operated in the reflection mode with Cu-K radiation (35 kV; 30 mA) and a diffracted beam monochromator, using a step scan mode with a step of 0.075° at 4 s per step. Diffraction patterns of both anatase and rutile TiO_2_ fibers at different flow rates were compared with a reference using the JCPDS database.

### 2.6. Photocatalytic Performance of TiO_2_ Fibers for NO Removal

The photocatalytic activities of TiO_2_@C1, TiO_2_@C2, TiO_2_@C3, and TiO_2_@C0 for the removal of NO gas molecules were assessed based on ISO 22197-1:2016. Hollow TiO_2_ microfiber (TiO_2_@C1, TiO_2_@C2, and TiO_2_@C3) or solid (TiO_2_@C0) TiO_2_ fiber (5 cm × 10 cm) samples were placed in the middle of two plain glasses (5 cm × 10 cm) of non-photocatalytic blank samples in a photoreactor. Each sample was irradiated with UV-A light (10 W m^−^^2^) using a UV lamp system, consisting of two 6 W lamps, placed over the photoreactor, with an emission peak at 365 nm. An NO_x_ analyzer (T-API, T200, San Diego, CA, USA) was used to measure nitrate concentrations at 1 min intervals. Under UV light irradiation, the NO gas flowed at a rate of 3 L min^−^^1^ containing 1 ppm_v_ of NO in air with 50% relative humidity at 25 °C. The concentration of NO in the outlet stream was monitored for 20 min before the light was switched on and afterward during the UV irradiation for 60 min.

## 3. Results and Discussion

### 3.1. Morphology of TiO_2_@C1, TiO_2_@C2, TiO_2_@C3, and TiO_2_@C0

**Figure 2:**
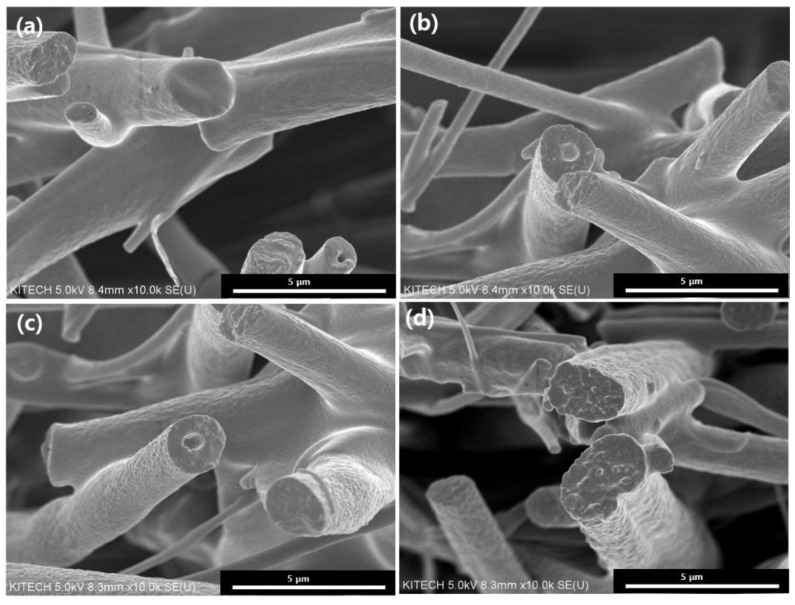
SEM images of hollow and solid TiO_2_ fibers before calcination: (**a**) TiO_2_@C1, (**b**) TiO_2_@C2, (**c**) TiO_2_@C3, and (**d**) TiO_2_@C0.

**Figure 3:**
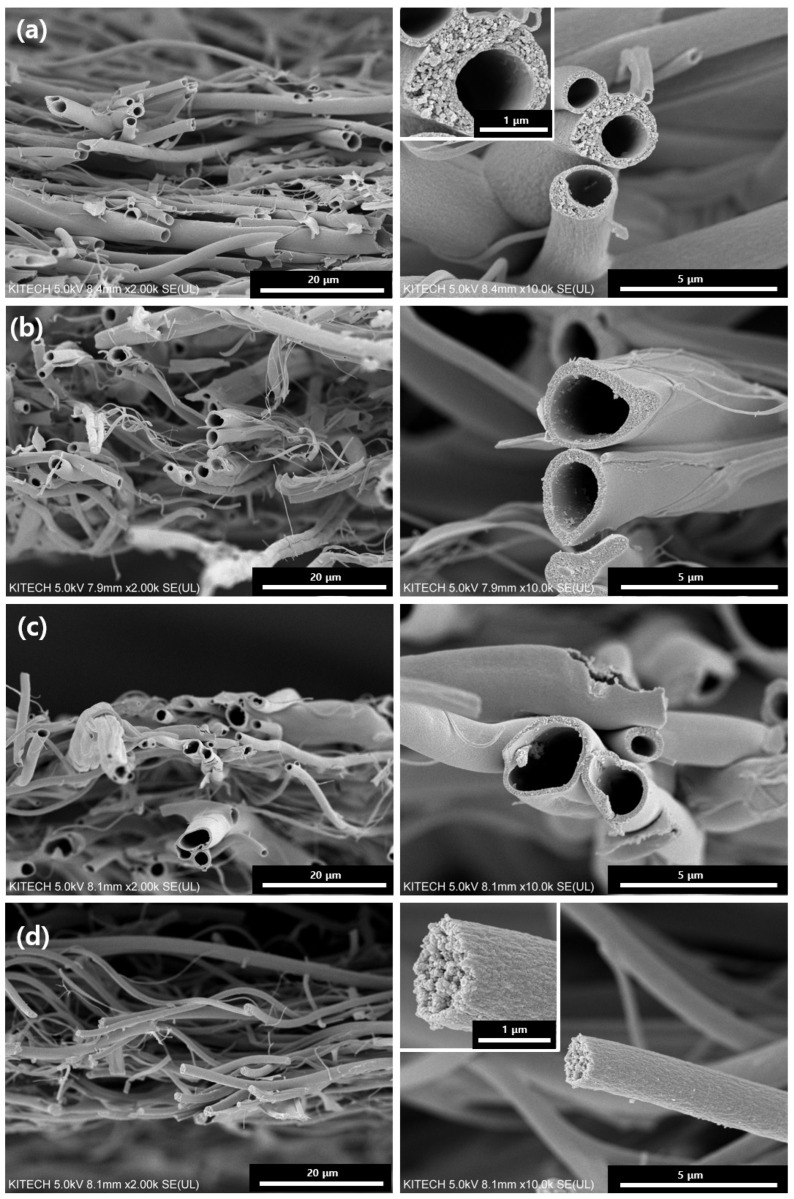
SEM images of hollow and solid TiO_2_ fibers after calcination: (**a**) TiO_2_@C1, (**b**) TiO_2_@C2, (**c**) TiO_2_@C3, and (**d**) TiO_2_@C0.

Figure 2 and Figure 3 show the synthesized TiO_2_ fibers before and after calcination, respectively. The synthesized TiO_2_ fibers have smooth surfaces without a hole inside. After calcination of the synthesized TiO_2_ fibers, they display a reduction in the average fiber diameter. The removal of the polymer (PVP and paraffin oil) by calcination shows a decrease in fiber diameter from about a few hundred nanometers, as shown in the SEM images in Figure 3. The average diameters of the core and sheath as well as the wall thickness of TiO_2_ fibers were measured at randomly selected areas of SEM images. The hollow core corresponds to the vacancy of the core paraffin oil emulsion. In Table 2, the TiO_2_@C1 has a core and sheath with average diameters of 1.45 and 1.81 µm, respectively, with a wall thickness of 0.26 µm. For TiO_2_@C2, the average diameters of the core and sheath are 1.80 and 2.09 µm, respectively, with 0.38 µm wall thickness. TiO_2_@C3 displays core and sheath diameters of 2.83 and 3.94 µm, respectively, with a wall thickness of 0.53 µm, while TiO_2_@C0 has an average fiber diameter of 3.95 µm without the core. Increasing the flow rate of the core paraffin fluid from 1 to 3 mL h^−1^ increased the core diameter from 0.26 to 0.53 µm and the outer diameter from 1.81 and 3.94 µm. Di et al. reported that hollow fibers electrospun with increased inner liquid flow rates of 1.0, 3.0, and 5.0 mL h−1 showed increased inner diameter from 0.9 to 1.90 µm [14]. Another study reported that decreasing the flow rate resulted in thicker hollow fibers because there was deficient outer solution to enclose the outer shell continuously [15]. Furthermore, the core flow rate was very slow, resulting in the outer layer being too thick due to less shear stress [13]. In contrast, a high core flow rate would make the wall thinner and decrease the stability of the electrospinning process because there may not be sufficient outer fluid to effectively capture the inner contents, leading to their leakage. Therefore, an appropriate flow rate is a critical variable for fabricating hollow fibers.

The flow rate of the core is an important factor in modifying the morphology of hollow TiO_2_ fibers, in terms of the core and outer diameter size. The higher the flow rate, the thinner the wall layer and the larger the core and outer diameters [16].

### 3.2. BET Analysis of TiO_2_@C1, TiO_2_@C2, TiO_2_@C3, and TiO_2_@C0

**Figure 4:**
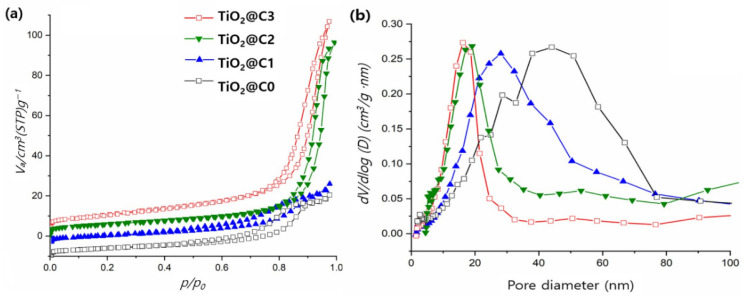
(**a**) BET plots for N_2_ adsorption/desorption isotherm, and (**b**) pore diameter distribution plots obtained using BJH plots of TiO_2_@C1, TiO_2_@C2, TiO_2_@C3, and TiO_2_@C0.

BET and BJH plots of TiO_2_@C1, TiO_2_@C2, TiO_2_@C3, and TiO_2_@C0 at 77 K are presented in Figure 4a,b. TiO_2_@C2 and TiO_2_@C3 showed a relatively narrow distribution of TiO_2_@C0, with values ranging from 5 to 30 nm. The order of the overall specific surface area is as follows: TiO_2_@C3 > TiO_2_@C2 > TiO_2_@C1 > TiO_2_@C0. The specific surface area of TiO_2_@C3 was approximately 51.2 m^2^ g^−1^. The increase in the core flow rate from 0 to 3 mL h^−1^ results in a corresponding increase in the specific surface area from 16.01 to 51.28 m^2^ g^−1^ and a decrease in the average pore diameter from 28.9 to 11.1 nm, as listed in Table 2. TiO_2_@C0 has the lowest specific surface area of 16.01 m^2^ g^−1^ and the highest average pore diameter size of 28.9 nm owing to the solid (non-hollow) structure of TiO_2_@C0. Previous studies have reported various advantages of hollow TiO_2_ fibers owing to their mesoporous walls and unique hierarchical pore structure [8]. This hollow structure helps to improve the efficiency of air mass transport, which leads to a larger specific surface area and porosity [17,18].

Hou et al. developed hollow TiO_2_ fibers with a BET surface area of ~27.2 m^2^ g^−1^ and an average pore diameter of 38 nm. However, our fabrication methods resulted in significantly higher specific surface area (~2 times higher than the aforementioned value), which provides more active surface sites for the adsorption of reactive molecules, resulting in more prominent photocatalytic effects [19]. The results suggest that hollow TiO_2_ fibers (TiO_2_@C3) prepared via the proposed method might exhibit good photocatalytic activities.

### 3.3. XRD Analysis of TiO_2_@C1, TiO_2_@C2, TiO_2_@C3, and TiO_2_@C0

**Figure 5:**
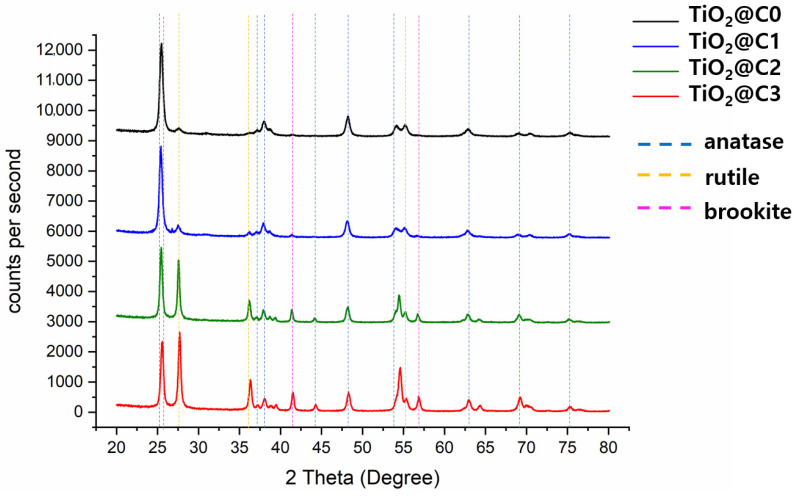
XRD spectra of TiO_2_@C1, TiO_2_@C2, TiO_2_@C3, and TiO_2_@C0.

After calcination, TiO_2_@C1, TiO_2_@C2, TiO_2_@C3, and TiO_2_@C0 contained anatase, brookite, and rutile phases. Generally, TiO_2_ has three representative crystalline phases, anatase, brookite, and rutile phases. TiO_2_ is most likely to be a mixture of the aforementioned phases rather than the pure anatase, brookite, or rutile structure; thus, quantitative analysis is significantly important for analyzing the variations in photocatalytic activities. Figure 5 and Table 3 present the XRD data of developed TiO_2_@C1, TiO_2_@C2, TiO_2_@C3, and TiO_2_@C0 in the 2θ range of 20° to 80° according to standard JCPDS card No. 21-1272. The anatase reflections dominated the reflection patterns, while rutile was present as well. All the diffraction peaks (blue dot lines) at 25.25°, 37.80°, 38.50°, 48.05°, 53.9°, 55.05°, 62.65°, 68.85°, 70.30°, 75.05°, and 76.10° could be well indexed as pure anatase phases. The brookite presents diffraction peaks (pink dot lines) at 2θ = 25.3, 27.7, 36.2, 42.3, 55.2, and 57.2° [20]. TiO_2_@C3 and TiO_2_@C0 displayed an increase in the rutile phase content and showed diffraction peaks (yellow dot lines) at 27°, 36°, and 55°, corresponding to the crystalline region of TiO_2_ [21]. The XRD results (Table 3) show that crystalline TiO_2_@C1 consists of 53.5% of the anatase phase, 7.2% of the rutile phase, and 39.3% of the brookite phase. TiO_2_@C2 and TiO_2_@C3 showed an increased rutile phase content of 11.8% and 24.3%, respectively, while TiO_2_@C0 had 60.1% of the anatase phase and 9.5% of the rutile phase. XRD data show that the increased core flow rate results in the higher composition of the anatase form and lower content of the rutile phase in the structure. TiO_2_ has three major stable polymorphs, namely, anatase, rutile, and brookite. Among them, anatase structure generally shows the highest photocatalytic activity, with certain crystallographic planes of anatase being particularly reactive [22]. Anatase transforms to the brookite or rutile phase at temperatures below 600°. A higher transformation rate of anatase to brookite, as compared with that of anatase to rutile, is observed and explained by the increased surface area of TiO_2_@C3, resulting from their aggregation, which act as sites for the rutile nucleation [23]. Upon heating, which is usually accompanied by a coarsening of the crystals, the crystal growth leads to alterations of phase stability [23]. The band gaps of anatase, rutile, and brookite are 2.13, 1.86, and 2.38 eV, respectively. Anatase is an indirect band gap semiconductor. In contrast, both rutile and brookite belong to the direct band gap semiconductor category [24]. It has been reported that a similar composition mixture of anatase and rutile crystalline phases of TiO_2_ exhibited significantly higher photocatalytic activity than the pure anatase phase [8,25]. The primary reason is the enhanced charge transfer triggered by the energy gap between the band edges of crystalline phases of TiO_2_ [8,26]. These photocatalysts show varying anatase/rutile ratios depending on the conditions of the core flow rate. Furthermore, the developed TiO_2_@C3 consists of a mixture of anatase/rutile (~1:1 ratio) crystalline phases, thereby exhibiting better photocatalytic performance than TiO_2_@C1, TiO_2_@C2, and TiO_2_@C0, as evident from our results.

### 3.4. Photocatalytic Performance of NO Removal

**Figure 6:**
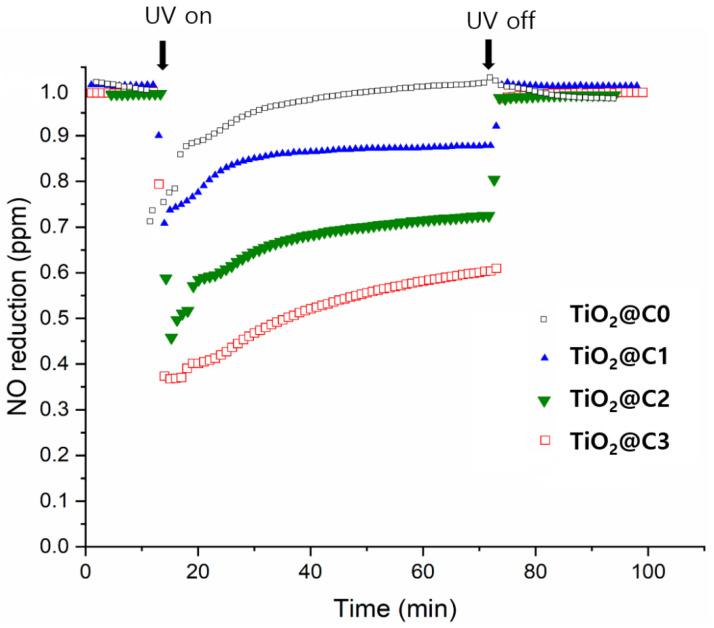
Photocatalytic performance of TiO_2_@C1, TiO_2_@C2, TiO_2_@C3, and TiO_2_@C4 for NO removal.

Figure 6 shows the NO removal performance of TiO_2_@C1, TiO_2_@C2, TiO_2_@C3, and TiO_2_@C0, which changes significantly with on-off UV lamp irradiation. To confirm the effectiveness of UV irradiation for triggering photocatalytic reaction, denitrification experiments were performed under UV irradiation (turning on UV lamp) and darkness (turning off UV lamp) conditions. The results show that the denitrification efficiency in the dark was ~0.01% during the initial 10 min. Moreover, under UV lamp irradiation, the NO removal (%) for TiO_2_@C1, TiO_2_@C2, TiO_2_@C3, and TiO_2_@C0 showed a rapid increase up to 66.2%, 56.8%, 33.5%, and 31.2%, respectively. When the UV lamp was turned off, the removal efficiency of NO decreased rapidly in the dark, reaching 1 ppm of NO concentration at 70 min and maintaining the NO concentration constant without photocatalytic activity. This implies a significant photocatalytic effect of UV irradiation on NO removal.

The degradation of NO gas molecules (1 ppm) was achieved in 60 min under light irradiation in the presence of TiO_2_@C1, TiO_2_@C2, TiO_2_@C3, and TiO_2_@C0. The highest efficiency was observed for the photocatalyst TiO_2_@C3, containing a mixture of 41.12% and 58.65% of rutile and anatase phases with minimum impurities, resulting in enhanced charge transfer owing to the energy gap between the two crystalline phases [8,26]. The highest BET specific surface area of 51.28 m^2^ g^−1^ leads to more active sites and higher capacity at the hollow TiO_2_ fiber surface for NO adsorption, resulting in more prominent photocatalytic effects. Amano et al. reported that the surface area and photocatalytic activity or capacity for reactant adsorption had a proportional relationship [27]. Therefore, TiO_2_@C3 is an efficient photocatalyst for air purification.

## 4. Conclusions

Herein, we demonstrated a facile method to fabricate easy-to-handle hollow TiO_2_ fibers as photocatalysts for air purification, particularly for denitrification. The hollow TiO_2_ fibers were fabricated using different core flow rates, ranging from 0 to 3 mL h^−1^, with a fixed outer flow rate of 8 mL h^−1^. The obtained TiO_2_ fibers showed mesoporous walls and a unique hierarchical pore structure. They were composed of mixed anatase and rutile phases with distinct hollow features. However, depending on the core flow rate, the fiber morphology changed in terms of the outer and core diameter sizes, wall thickness, BET specific surface area, and crystalline phase content as well as photocatalytic activity. Results show that TiO_2_@C3 had the largest core and outer diameters that improve the air mass transport. Furthermore, among the prepared photocatalysts, TiO_2_@C3 exhibited a balanced anatase/rutile phase ratio as well as the highest BET specific surface area of 51.28 m^2^ g^−1^ and smallest pore size of 11.1 nm, offering more active sites for NO removal and resulting in the most effective denitrification up to 66.2%.

This study provides a comprehensive understanding and an ideal design for the fabrication of hollow TiO_2_ fibers in terms of the crystalline phase composition as well as hollow and mesoporous structures for the desired photocatalytic performance. Our methodology presents a facile approach to fabricate hollow architecture for various applications including surface coating, electronics, biomedicine, sensing, and water and air purification.

## Figures and Tables

**Table 1:** Electrospinning conditions and sample codes of hollow and solid (non-hollow) TiO_2_ fibers.

	Flow Rate (mL h^−1^)			
Outer	8			
Core	1	2	3	0
Sample #	TiO_2_@C1	TiO_2_@C2	TiO_2_@C3	TiO_2_@C0

**Table 2:** Physical properties of TiO_2_@C1, TiO_2_@C2, TiO_2_@C3, and TiO_2_@C0.

	TiO_2_@C1	TiO_2_@C2	TiO_2_@C3	TiO_2_@C0
Average sheath diameter (µm)	1.81 (±0.8)	2.09 (±0.88)	3.94 (±1.43)	3.95 (±1.44)
Average core diameter (µm)	1.51 (±0.37)	1.80 (±1.01)	2.83 (±1.28)	-
Wall thickness (µm)	0.53 (±0.30)	0.38 (±0.10)	0.26 (±0.10)	-
BET surface area (m^2^ g^−1^)	33.04	40.73	51.28	16.01
Average pore diameter (nm)	18.8	11.3	11.1	28.9

**Table 3:** Relative composition of crystalline phases and NO removal (%) of TiO_2_@C1, TiO_2_@C2, TiO_2_@C3, and TiO_2_@C0.

	TiO_2_@C1	TiO_2_@C2	TiO_2_@C3	TiO_2_@C0
Anatase (%)	53.5	15.3	25	60.1
Rutile (%)	7.2	11.8	24.3	9.5
Brookite (%)	39.3	72.9	50.7	30.3
NO removal (%)	33.5	56.8	66.2	31.2

## Data Availability

Not applicable.
